# A prospective study comparing highly qualified Molecular Tumor Boards with AI-powered software as a medical device

**DOI:** 10.1007/s10147-024-02684-z

**Published:** 2024-12-23

**Authors:** Hideaki Bando, Yoichi Naito, Tomoyuki Yamada, Takao Fujisawa, Mitsuho Imai, Yasutoshi Sakamoto, Yusuke Saigusa, Kouji Yamamoto, Yutaka Tomioka, Nobuyoshi Takeshita, Kuniko Sunami, Megumi Futamura, Chiemi Notake, Satoko Aoki, Kazunori Okano, Takayuki Yoshino

**Affiliations:** 1 Translational Research Support Office, Division of Drug and Diagnostic Development Promotion, Department for the Promotion of Drug and Diagnostic Development, National Cancer Center Hospital East, 6-5-1 Kashiwanoha, Kashiwa, Chiba 277-8577 Japan; 2 Division of Data Science, Department for the Promotion of Drug and Diagnostic Development, National Cancer Center Hospital, East 6-5-1 Kashiwanoha, Kashiwa, Chiba 277-8577 Japan; 3 Department of Gastroenterology and Gastrointestinal Oncology, National Cancer Center Hospital East, 6-5-1, Kashiwanoha, Kashiwa, Chiba 277-8577 Japan; 4 Department of General Internal Medicine, National Cancer Center Hospital East, 6-5-1 Kashiwanoha, Kashiwa, Chiba 277-8577 Japan; 5 Genomedia Inc, 4-1-4 Hongo, Bunkyo-ku, Tokyo 113-0033 Japan; 6 Department of Head and Neck Medical Oncology, National Cancer Center Hospital East, 6-5-1 Kashiwanoha, Kashiwa, Chiba 277-8577 Japan; 7 Department of Genetic Medicine and Services, National Cancer Center Hospital East, Chiba, 277-8577 Japan; 8 Department of Biostatistics, Yokohama City University, 3-9 Fukuura, Kanazawa-Ku, Yokohama, Kanagawa 236-0004 Japan; 9 Department for the Promotion of Medical Device Innovation, National Cancer Center Hospital East, 6- 5-1 Kashiwanoha, Kashiwa, Chiba 277-8577 Japan; 10 Department of Laboratory Medicine, National Cancer Center Hospital, Tokyo, 104-0045 Japan

**Keywords:** Comprehensive genomic profiling, Molecular Tumor Board, Cancer precision medicine, AI-powered diagnostic tools

## Abstract

**Background:**

The implementation of cancer precision medicine in Japan is deeply intertwined with insurance reimbursement policies and requires case-by-case reviews by Molecular Tumor Boards (MTBs), which impose considerable operational burdens on healthcare facilities. The extensive preparation and review times required by MTBs hinder their ability to efficiently assess comprehensive genomic profiling (CGP) test results. Despite attempts to optimize MTB operations, significant challenges remain. This study aims to evaluate the effectiveness of QA Commons, an artificial intelligence-driven system designed to improve treatment planning using CGP analysis. QA Commons utilizes a comprehensive knowledge base of drugs, regulatory approvals, and clinical trials linked to genetic biomarkers, thereby enabling the delivery of consistent and standardized treatment recommendations. Initial assessments revealed that the QA Commons’ recommendations closely matched the ideal treatment recommendations (consensus annotations), outperforming the average results of MTBs at Cancer Genomic Medicine Core Hospitals.

**Methods:**

A clinical performance evaluation study will be conducted by comparing the QA Commons’ treatment recommendations with those of the Academia Assembly, which includes medical professionals from the Cancer Genomic Medicine Core and Hub Hospitals. One hundred cases selected from the “Registry of the Academia Assembly,” based on defined inclusion and exclusion criteria, will be analyzed to assess the concordance of recommendations.

**Conclusion:**

The expected outcomes suggest that QA Commons could reduce the workload of MTB members, standardize the quality of MTB discussions, and provide consistent outcomes in repeated patient consultations. In addition, the global expansion of QA Commons could promote worldwide adoption of Japan’s pioneering precision oncology system.

## Introduction

### Comprehensive genomic profiling (CGP) testing and Molecular Tumor Boards (MTBs) for advanced solid tumors

Recent advancements in molecular testing, notably next-generation sequencing (NGS), have propelled cancer precision medicine to utilize individual genetic mutations to customize cancer treatment. A key development is the use of NGS for CGP, which facilitates the simultaneous analysis of many genes for direct treatment [1]. The introduction of cancer precision medicine in Japan, highlighted by the 2018 approval of FoundationOne® CDx and the OncoGuide NCC Oncopanel System, represents a significant breakthrough in this domain [2].

The process of converting CGP results into actionable treatment plans necessitates clinical annotations that require a broad spectrum of expertise, including oncology, pharmacotherapy, genetic medicine, and bioinformatics. In Japan, the critical task of interpreting CGP results is undertaken by MTBs, composed of specialists from various relevant fields [3]. To facilitate the implementation of cancer precision medicine, the Ministry of Health, Labor and Welfare (MHLW) has established “Cancer Genomic Medicine Core Hospitals (Core Hospitals)” and “Cancer Genomic Medicine Hub Hospitals (Hub Hospitals)” [2, 3]. As of August 2024, 13 Core Hospitals and 32 Hub Hospitals have been designated for this purpose. The involvement of MTBs at these centers is essential for the approval and performance of CGP tests under health insurance coverage [4].

A major challenge for MTBs in precision medicine is the significant operational burden they impose on healthcare facilities. The Japanese Society of Medical Oncology (JSMO) survey of Core Hospitals revealed that MTBs generally convene weekly, requiring extensive pre-meeting work-up of 35 h in preparation and an additional 2 h for further review. The substantial time and effort required pose a bottleneck, limiting their capacity to only approximately 1800 cases monthly across all facilities [4]. Thus, optimizing and accelerating MTB operations is vital for expanding access to CGP tests, ensuring timely and appropriate treatment decisions, facilitating participation in clinical trials, and, ultimately, advancing Japan’s cancer precision medicine. Another issue with MTBs is the one-time consultation per patient, despite potential changes in treatment recommendations due to new drug approvals and the start of clinical trials. A delay between the generation of MTB recommendations and actual treatment considerations can result in outdated advice [5, 6].

### QA Commons

**Fig. 1:**
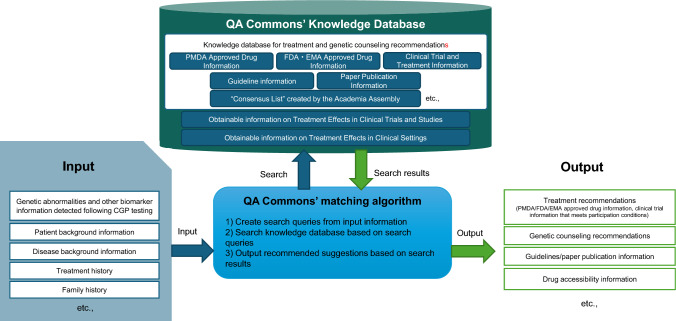
Overview of QA Commons. This diagram provides a schematic representation of QA Commons. The system receives input information, including results from CGP testing, patient background, disease background, treatment history, and family history. Using a matching algorithm that references QA Commons’ knowledge database, the system generates output reports. These reports include treatment recommendations, genetic counseling suggestions, guideline and publication references, and drug accessibility information

Genomedia Inc. (GMI) is developing QA Commons (Fig. 1), an artificial intelligence (AI)-based system that supports treatment planning based on CGP analysis. To offer personalized treatment recommendations, QA Commons integrates a knowledge database with drug approval and clinical trial information related to genetic biomarkers, along with patient data input and output functionalities. Originally supported by the Ministry of Economy, Trade, and Industry’s New Energy and Industrial Technology Development Organization in 2017, the project aimed to construct a rapid interpretation system for cancer gene variations using the latest information. It was further developed by the Japan Agency for Medical Research and Development. This project involved collaboration with an MTB of multidisciplinary specialists across institutions and aimed to create a comprehensive system and training program. GMI’s ongoing development of QA Commons signifies its commitment to improving precision oncology treatments.

The primary function of the QA Commons is to recommend optimal therapies based on identified genomic abnormalities. In addition, it has the capability to detect and present potential secondary findings associated with these abnormalities. By incorporating additional data, such as family history, the system can suggest the possibility of hereditary cancers. Based on these insights, the software is also equipped to propose referrals to specialized genetic counseling for further evaluation.

A preliminary study conducted by the “Yoshino subgroup,” as part of a research initiative focused on developing educational and training methods for professionals in cancer precision medicine, evaluated the performance of QA Commons. They evaluated the concordance between recommendations generated by QA Commons and “Consensus annotations”—ideal treatment recommendations derived from the collective input of key physicians at Core Hospitals—using 50 mock cases. The study, conducted in collaboration with the MTBs of Core and Hub Hospitals, aimed to assess the alignment between AI-generated recommendations and “Consensus annotations.” The results demonstrated that QA Commons achieved a favorable 84% agreement rate with “Consensus annotations.” In comparison, the overall agreement rate between hospital MTBs and “Consensus annotations” was 62%, with individual facility rates ranging from as low as 48% to as high as 86%. Thus, the performance of QA Commons matched that of the highest-performing facility [5, 6].

### Attempts to improve and harmonize MTBs

The “Yoshino subgroup,” committed to advancing cancer precision medicine in Japan, conducted research focused on developing and training specialized personnel for MTBs at Core and Hub hospitals. This initiative was supported by a Health and Labor Sciences Research Grant. As part of this effort, key physicians from each Core and Hub hospital’s MTB collaborated to create “Consensus annotations” for simulated cases, assess the consistency of MTB recommendations across different hospitals, and implement educational programs, namely the “Ekipane Program” and “Ekipane Dojo.” These programs were conducted in partnership with the JSMO.

Findings from the “Yoshino Subgroup” revealed a 62% overall agreement rate between Core Hospital MTBs and “Consensus annotations,” highlighting the variability in treatment recommendations across facilities. High agreement rates (88 − 100%) were observed for gene alterations with high evidence levels (A/B/R) [7], whereas gene alterations with lower evidence levels (C/D/E) [7] demonstrated significantly lower agreement rates (18 − 30%). The reports also highlighted inconsistencies in treatment recommendations for similar CGP test results within the same MTBs, emphasizing the critical need for standardized approaches in cancer precision medicine.

Following the dissolution of the “Yoshino Subgroup” in 2021, the “Academia Assembly” was established to elevate the standards of cancer genomic medicine in Japan. Building on the expertise of Yoshino Subgroup members, this new initiative includes medical professionals from Core and Hub hospitals, as well as experts from clinical trial centers specializing in oncology, pathology, genetics, and genetic counseling. The “Academia Assembly” facilitates secure collaboration and data sharing among these specialists to refine and continuously update the lists of matched therapies and clinical trials in cancer precision medicine, collectively referred to as the “Consensus List.” The “Consensus List” is updated monthly and will be shared, under contractual agreements, with technology companies developing AI-powered MTBs. These AI-powered MTBs will incorporate the “Consensus List” into their algorithms, using it to generate highly accurate treatment recommendations.

In addition, the “Academia Assembly” has launched a comprehensive study to assess the effectiveness of consensus-based treatment annotations, known as “Consensus annotations,” which are vital for advancing precision medicine. This initiative aligns with the broader objective of integrating AI into cancer care and encouraging AI companies to develop programs for therapeutic planning. The integrity of the data used in these efforts is rigorously maintained through external audits conducted by the Japanese Society of Clinical Oncology, ensuring the reliability and impact of their contributions to cancer precision medicine.

## Patients and methods

### Clinical performance study for QA Commons

#### Study overview and inclusion and exclusion criteria

We are currently organizing a clinical performance study for QA Commons that will evaluate the concordance between the treatment recommendations made by QA Commons and those suggested by the “Academia Assembly.” This study will focus on specific cases that meet predefined inclusion and exclusion criteria. These cases will be selected from the “Multicenter Joint Case Registry Study in the Academia Assembly (Registry of the Academia Assembly)” based on available clinical data and CGP results. The primary objective of this study is to evaluate the clinical performance of the QA Commons by measuring the agreement between its treatment recommendations and those proposed by the “Academia Assembly.” The key eligibility criteria include patients aged > 18 years who have undergone CGP testing within a clinical setting and provided informed consent for participation and the secondary use of their information in the registry study without any subsequent withdrawal or refusal.

#### Statistical considerations

In this study, the total planned number of cases is set at 100, consisting of 75 cases with evidence levels A/B/R and 25 cases with evidence levels C/D/E. The rationale for this distribution is to test the non-inferiority of concordance rates for high evidence level (A/B/R) cases and the superiority of concordance rates for low-evidence level (C/D/E) cases when compared to the treatment recommendations provided by QA Commons. This approach aims to rigorously evaluate the performance of QA Commons across varying levels of evidence strength, ensuring a comprehensive assessment of its clinical utility.

In the previous study conducted by the “Yoshino subgroup” at core hospitals, the overall average concordance rate with “Consensus annotation” was 62% (range: 48 − 86%), with concordance rates for gene alteration of 88 − 100% for high-evidence levels (A/B/R) and 18 − 30% for low-evidence levels (C/D/E). The concordance rate for the A/B/R cases was 89% (range: 78 − 100%). Another study using the QA Commons prototype reported concordance rates of 91.7% (95% CI, 61.5 − 99.8%) for high-evidence level cases and 87.1% (95% CI, 70.2 − 96.4%) for low-evidence level cases. To confirm the robustness of this product, two evaluations were conducted, and the QA Commons treatment recommendations were deemed acceptable only if both evaluations met the criteria. The sample size was set to ensure an overall detection power of 80%.

For these reasons, the expected concordance rate for QA Commons in the high-evidence level (A/B/R) case group was set at 93%, with a reference value of 89%, with the lowest concordance rate of 78% for MTB (non-inferiority margin of 11%). Considering potential fluctuations in evidence levels, dropouts, and unevaluable cases, the target sample size was set at 75. For the superiority of QA Commons in the low-evidence level (C/D/E) case group, with an expected concordance rate of 80% and a threshold concordance rate of 40%, a sample size of 10 cases was sufficient to ensure detection power. Considering potential fluctuations in evidence levels, dropouts, and unevaluable cases, the target sample size was set at 25 cases.

The threshold concordance rates of 89% and 40% were deemed appropriate for the high- and low-evidence level case groups, respectively, based on consensus during the “Ekipane Program” at Core hospitals and consultations with the Cancer and Disease Control Division of the MHLW. Therefore, in this study, performance will be evaluated in 100 cases: 75 and 25 cases in the high- and low-evidence level groups, respectively.

#### Case selection

**Fig. 2:**
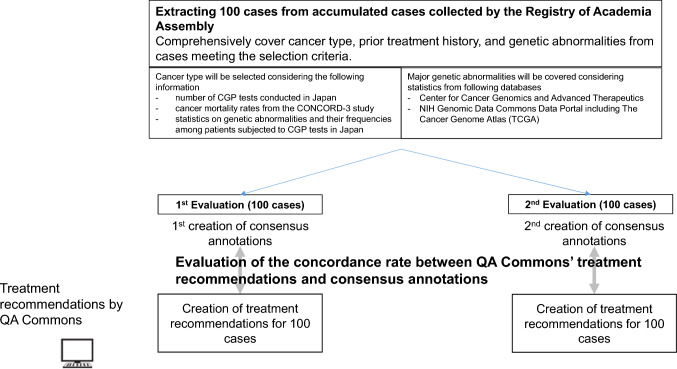
Trial design overview for evaluating QA Commons treatment recommendations. This figure illustrates the trial design for evaluating QA Commons’ treatment recommendations. A total of 100 cases will be selected from the registry of the Academia Assembly, ensuring comprehensive coverage of cancer types, prior treatment histories, and genetic abnormalities. Consensus annotations will be generated twice within an interval of a few months, and the concordance rate with QA Commons’ treatment recommendations will be assessed

The study coordinating office will ask the “Academia Assembly” to extract 100 cases from the registered cases in the “Registry of the Academia Assembly” that satisfy the following criteria, ensuring that the distribution of cancer types and genetic mutations reflects real-world clinical settings and covers the cancer types and genetic mutations necessary for clinical performance evaluation by QA Commons. (Fig. 2.)

1. The selection of cases will include a variety of cancer types, including colorectal, gastric, esophageal, hepatocellular, pancreatic, biliary tract, breast, lung, prostate, ovarian, cervical, and malignant melanomas; brain tumors; head and neck, thyroid, bone, and soft tissue tumors; and adrenal cortical cancers. The breakdown of cancer types will be determined based on the number of CGP tests conducted in Japan, cancer mortality rates from the CONCORD-3 study [8], and statistics on genetic abnormalities and their frequencies among patients who have undergone CGP tests in Japan, as collected by the “Academia Assembly” through surveys conducted at the Core and Hub hospitals.
2. As CGP tests are generally conducted on patients with solid tumors for whom no standard treatment exists or who have completed standard treatments (including those expected to complete treatment), pretreatments are commonly shared. However, for major cancer types such as colorectal, gastric, breast, and non-small cell lung cancers, there are multiple salvage line treatment options, and not all approved drugs are used. Therefore, when selecting cases, the major patterns of prior treatment will be covered from a clinical perspective.
3. We will cover the major genetic mutations for each cancer type. This includes, as a principle, tumor-agnostic genetic mutations (such as Microsatellite Instability-High, Tumor Mutational Burden-High, and *NTRK* fusion genes). The breakdown of genetic mutations will be determined based on the frequency of genetic mutations in specific diseases, as referenced from the Cancer Genome Information Management Center and the National Institutes of Health Genomic Data Commons Data Portal, including The Cancer Genome Atlas [9].

#### Study outline

**Fig. 3:**
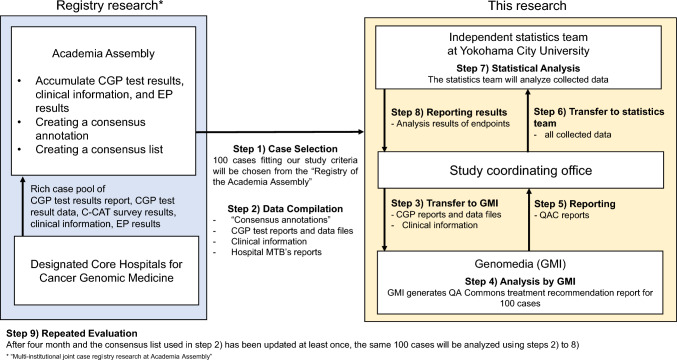
Outline of the study procedures. This figure depicts the study procedures, beginning with the selection of 100 cases from the “Registry of the Academia Assembly.” The data are compiled and sent to Genomedia Inc. (GMI) for analysis, resulting in the generation of QA Commons’ treatment recommendation reports. These reports are then evaluated by a statistics team. After 4 months, the process will be repeated to verify consistency in the findings

This clinical performance study will be conducted according to the following procedures:

- **Step 1. Case selection**: From the registry of the “Academia Assembly,” 100 cases that fit the study criteria will be selected.
- **Step 2. Data compilation**: The “Academia Assembly” prepares “Consensus annotations” for these cases, which, along with CGP test reports, data files, clinical information, and the MTB reports of the various hospitals, are securely sent to the study coordinating office.
- **Step 3. Transfer to GMI**: The study coordinating office forwards CGP test reports, data files, and clinical information to the GMI using a secure system.
- **Step 4. Analysis by GMI**: Upon receiving the data, GMI will input the information into QA Commons, referencing QA Commons’ Knowledge Database that will be created by incorporating information from the “Consensus List” in advance. QA Commons will then generate recommendation reports for the 100 cases.
- **Step 5. Reporting**: These reports are sent back to the study coordinating office.
- **Step 6. Transfer to statistics team**: The study coordinating office sends all collected data to the statistics team for evaluation of the study’s endpoints.
- **Step 7. Statistical analysis:** The statistics team will analyze the collected data and evaluate the study’s endpoints.
- **Step 8. Reporting results**: The statistical team reports the analysis results of the endpoints to the study coordinating office.
- **Step 9. Repeated evaluation**: After 4 months of initial analysis and the “Consensus list” used in step 4 has been updated at least once, the procedure from steps 2 to 8 is repeated with the same 100 cases to verify the consistency of the findings. (Fig. 3.)

## Discussion

With the rapid progress in the clinical development of targeted therapies and the accumulation of new findings related to genetic information, it is essential to adopt computational approaches to manage these processes [10]. QA Commons is an AI program designed to enhance treatment planning using CGP analysis. It integrates a knowledge database containing detailed information on drugs, approvals, and clinical trials associated with genetic biomarkers. QA Commons aims to expedite the reporting of cancer genomic variations and improve precision oncology treatments through a system incorporating multidisciplinary expertise. A preliminary evaluation showed that the QA Commons’ recommendations surpassed the average alignment of hospital MTBs and matched the performance of the highest-performing MTB. This underscores the potential of QA Commons to significantly advance the field of cancer precision medicine.

In Japan, genomic medicine is uniquely linked to insurance payments and the requirement for MTBs to review every case. In addition, there is a mandate to register clinical information with the Center for Cancer Genomics and Advanced Therapeutics, which places a considerable workload on both Core and Hub hospitals [4]. To address these challenges, some amendments have been made to reduce the required number of MTB members and introduce a rapid evaluation process for cases with only evidence level A recommendations without suspicion of hereditary tumors. Plans are underway to expand the use of MTBs in cooperative hospitals. However, these measures do not provide fundamental solutions. AI-powered diagnostic programs can reduce the burden on MTB members. In addition, they offer the benefit of standardizing the quality of MTBs and providing consistent results for the same patient.

However, QA Commons has certain limitations that must be addressed. First, the planned clinical performance study includes 100 adult patients with cancer; hence, the clinical evaluation of QA Commons will be limited to a subset of adult patients with cancer. Consequently, its initial healthcare application will also be restricted to this patient population. Nonetheless, following statistical validation of its utility in this study and subsequent regulatory approval, QA Commons’ scope of application will be expanded. Future efforts will focus on incorporating additional cancer types, including pediatric cancers, as well as a broader range of genetic abnormalities and recommended therapies, ensuring comprehensive coverage and continuous improvement of its functionality.

Future approval and clinical implementation of QA Commons are expected to have a positive ripple effect on healthcare in Japan. This includes reducing the time and number of MTB sessions, which, in turn, reduces the workload of the medical staff. In addition, it has the potential to increase the number of CGP tests, a challenge previously caused by the limited number of Core and Hub hospitals. With MTBs being less burdened, freed-up time can be utilized to enhance overall medical services. Furthermore, adopting QA Commons International could pave the way for Japan’s genomic medicine system to expand globally, showcasing its innovative approach to healthcare.
